# Intelligent ZHENG Classification of Hypertension Depending on ML-kNN and Information Fusion

**DOI:** 10.1155/2012/837245

**Published:** 2012-06-03

**Authors:** Guo-Zheng Li, Shi-Xing Yan, Mingyu You, Sheng Sun, Aihua Ou

**Affiliations:** ^1^Department of Control Science and Engineering, Tongji University, Shanghai 201804, China; ^2^The Department of Clinical Epidemiology and The Cardiovascular Medicine of Chinese Medical, Guang Dong Provincial Hospital of Traditional Chinese Medicine, Guangzhou 510120, China

## Abstract

Hypertension is one of the major causes of heart cerebrovascular diseases. With a good accumulation of hypertension clinical data on hand, research on hypertension's ZHENG differentiation is an important and attractive topic, as Traditional Chinese Medicine (TCM) lies primarily in “treatment based on ZHENG differentiation.” From the view of data mining, ZHENG differentiation is modeled as a classification problem. In this paper, ML-kNN—a multilabel learning model—is used as the classification model for hypertension. Feature-level information fusion is also used for further utilization of all information. Experiment results show that ML-kNN can model the hypertension's ZHENG differentiation well. Information fusion helps improve models' performance.

## 1. Introduction

Hypertension is one of the major causes of heart cerebrovascular diseases. 25%–35% adults over the world have hypertension. There are over 972 million hypertension patients, of which 60%–70% are over 70 years old [1, 2]. With the fast development of electronic medical record (EMR) system, there exists a good accumulation of clinical cases about hypertension. As diagnostic knowledge and herb formula of Traditional Chinese Medicine (TCM) are mostly distilled from clinical practice, researches on these clinical cases may help promote the understanding toward TCM theory, make progress on the development of diagnosis technology, and also contribute to the objection and modernization of TCM.

ZHENG, also translated as syndrome, in TCM means a characteristic profile of all clinical manifestations that can be identified by a TCM practitioner. TCM lies primarily in “treatment based on ZHENG differentiation” [3]. Only after successful differentiation of ZHENG, can effective treatment of TCM be possible [4]. Traditionally, techniques of ZHENG differentiation are learned by successors of a particular TCM practitioner only and learning effect is always confined to the successors' personal talents. With the unprecedented growth of clinical data, this way is no longer proper, which makes it difficult to discover new knowledge from the data mountain. Data mining is a distinguished technology to track the underlying information. Many research works have been dedicated to TCM data mining [5–7], all of which indicate a promising future for auto differentiation of ZHENG in TCM.

In the field of data mining, differentiation of ZHENG is modeled as a classification problem. For traditional classification methods, every instance should have one and only one label. However, TCM diagnostic result usually consists of several ZHENG. In other words, one patient could have more than one ZHENG. Professionally, it is called multilabel data, the learning of which is a rather hot topic recently in the fields of data mining and machine learning. International workshops about multilabel learning are held in the recent three years, respectively, to promote the development of this topic [8, 9]. Multilabel learning has been applied to TCM by Liu et al. [7], who compared the performance of ML-kNN and kNN on a coronary heart disease dataset. Li et al. and Shao et al. proposed embedded multilabel feature selection method MEFS [10] and wrapper multilabel feature selection method HOML [11], respectively, to improve multilabel classification's performance on a coronary heart disease dataset.

One characteristic of TCM ZHENG differentiation is “fusion use of four classical diagnostic methods.” Inspection, auscultation and olfaction, inquiry and palpation are the four classical diagnostic methods in TCM. How to use information from these four diagnostic methods to make better ZHENG differentiation is an important research area in TCM field. Some theories of Traditional Chinese Medicine diagnosis even claim that only by using information from all the four classical diagnostic methods can we differentiate correctly the ZHENG [4]. And “fusion use of the four classical diagnostic methods” is treated as an important direction in computerization of TCM diagnosis [12]. In fact, it is called information fusion in the field of data mining. Therefore, fusion of information from different sources should be considered seriously in building ZHENG classification with multilabel learning techniques. Nowadays, no researchers have tried to bring techniques of information fusion into the field of multilabel learning. Wang et al. have done some work in TCM information fusion using traditional single-label methods, which mainly focus on the data acquisition and medical analysis on experiment results [12, 13]. But as described above, multilabel learning should be more appropriate for ZHENG classification. So more attention should be paid on the research of information fusion for multilabel learning.

In this paper, we try to build TCM ZHENG classification models on hypertension data using multilabel learning and information fusion. The rest of the paper is arranged as follows.  Section 2 describes materials and methods, including the data source, data preprocessing, feature-level information fusion, and ML-kNN. Experimental results and discussions are shown in Section 3. Finally Section 4 draws conclusions on this paper.

## 2. Materials and Methods

### 2.1. Data Source

The hypertension datasets used in this paper are from LEVIS Hypertension TCM Database. The data are from the in-patient, out-patient cases of Cardio Center, Cardiovascular Internal Department, Nerve Internal Department, and Medical Examination Center, and so forth in Guangdong Provincial Hospital of TCM in China during November 2006 to December 2008, as well as some cases from on-the-spot investigation in Li Wan District Community in Guangzhou of China during March 2007 to April 2007. With strict control measures, 775 reliable TCM hypertension clinical cases are recorded in this database. 148 features, including 143 TCM symptoms from inspection, auscultation and olfaction, inquiry and palpation, and 5 common indexes including gender, age, hypertension duration, SBPmax, and DBPmax, are investigated and collected in this database. It also stores the 13 labels (TCM ZHENG) of each case. Academic and noncommercial users may access it at http://levis.tongji.edu.cn/datasets/index_en.jsp. 

### 2.2. Data Preprocessing

According to the theory of TCM, the characteristics of the LEVIS Hypertension TCM Database, and our research target that evaluation of the performance of multilabel classification model on datasets with information from particular diagnostic methods only (we call them single-diagnosis datasets later) and on dataset with fusional information of all diagnostic methods (called fusional-diagnosis dataset), five single-diagnosis datasets are retrieved from the LEVIS Hypertension TCM Database. The information contained in each datasets is shown in Tables 1, 2, 3, 4, and 5, which comes, respectively, from inspection diagnosis, tongue diagnosis, inquiry diagnosis, palpation diagnosis, and other diagnoses. Analyzing the 775 cases, 4 cases are found to have empty value in one of the features mentioned above in the five tables. Thus, these 4 cases are removed from all the five single-diagnosis datasets to ensure smooth progress of the following tasks: information fusion and classification model building.

In the above data sets, we find some labels appear rarely, which will severely hurt severely performance of classification methods. We randomly choose part of the data set in this work. Firstly, labels are selected to decrease the degree of imbalance. In this case, we chose labels 6, 10, and 12, as they have the largest number of positive cases and multilabel method should predict at least 3 labels simultaneously. Secondly, cases are selected that are marked negative on all the selected labels to be the pending removable set, so that the entire positive cases in any label are preserved. Finally, randomly remove some cases from the pending removable set to decrease imbalance. Here, 500 cases are put into the pending removable set and 100 cases are selected from the set to form one dataset with remaining cases each time. So finally, we get five datasets and the performance of our model is evaluated according to the average performance on all datasets. The final used data set may be downloaded from: http://levis.tongji.edu.cn/datasets/htn-ecam.zip.

### 2.3. Feature-Level Information Fusion

In this work, we only discuss information fusion on the level of feature [14, 15]. Let *A* = {*a*_1_, *a*_2_,…,*a*_*n*_}, *B* = {*b*_1_, *b*_2_,…,*b*_*m*_}, *C*, *D*, *E* denote, respectively, the 5 feature vectors with different dimensions illustrated in Tables 1–5. The target is to combine these five feature sets in order to yield a new feature vector, *Z*, which would better represent the individual or help build better classification model [14]. Specifically, information fusion is accomplished by simply augmenting the information (feature) obtained from multiple diagnostic methods. The vector *Z* is generated by augmenting vectors *A* to *B*, *C*, *D*, and *E* one after the other. The concrete stages are described below:

1. *Feature Normalization*. The individual feature values of particular vectors, such as *a*_11_ and *b*_*m*2_, may exhibit significant variations both in their range and distribution. The goal of feature normalization is to modify the location (mean) and scale (variance) of the values to ensure that the contribution of each vector to the final vector *Z* is comparable. Min-max normalization techniques were used in this work. It computes the value *x*′ after normalization using the formula, *x*′ = (x − min⁡(Fx))/(max⁡(Fx) − min⁡(Fx)), where x and *x*′ denote, respectively, a feature value before and after normalization and Fx is the feature value set that contains all values of a specific feature. Normalizing all feature values via this method, we get the modified feature vectors *A*′, *B*, *C*′, *D*′, and *E*′.
2. *Feature Concatenation*. Augment the 5 feature vectors, which results in a new feature vector, *Z*′ = {*a*_1_′,…, *a*_*n*_′, *b*′_1_,…, *b*_*m*_′,…, *e*_1_′,…, *e*_*l*_′}.

### 2.4. Multilabel Learning: ML-kNN

As illustrated in Section 1, multilabel learning model is believed to be more suitable classification model for TCM clinical data. Specifically, we constructed models of the relationship between symptoms and ZHENG by means of the multilabel k-nearest neighbor (ML-kNN) algorithm [16] in this study. ML-kNN is a lazy multilabel learning algorithm developed on the basis of kNN algorithm, which regards an instance as a point in synthesis space. kNN's idea is to search for k training instances nearest to the testing instance, and then predict the label of the test instance according to the nearest instances' labels. Compared with other algorithms, advantage of kNN lies in its simpler training process, better efficiency, and competitive performance. Based on the theory of kNN, ML-kNN also aims to find k nearest instances for each test instance. But rather than judging labels directly by nearest instances, ML-kNN utilizes the “maximum a posteriori estimation” principle to determine the label set based on statistical information derived from the label sets of neighboring instances. The concrete steps are demonstrated below [7]:

1. calculate the conditional probability distribution of each instance associated to each label;
2. calculate the distance between the x_*i*_ test instance and the training instances; then find k nearest instances for x_*i*_. Repeat for each test instance;
3. according to the labels of k training instances and the conditional probability associated to each label, forecast the probability of the x_*i*_ instance and then acquire the forecast results (≥0.5 is taken here); Repeat for each test instance;
4. evaluate the forecast results according to multilabel evaluation criteria.

## 3. Results and Discussions

### 3.1. Experiment Setting and Procedure

Firstly, five single-diagnosis datasets are retrieved from LEVIS Hypertension TCM Database as illustrated in Section 2.1. Secondly, data preprocessing is conducted on all the five datasets as described in Section 2.2. Thirdly, feature-level information fusion mentioned in Section 2.3 is applied to the single-diagnosis datasets and yields fusional-diagnosis dataset. There are five single-diagnosis datasets and one fusional-diagnosis dataset. Fourthly, ML-kNN is used to train models and test models on all the 6 datasets with parameter k set to be 10; to better reveal performance of models, 10-fold cross-validation is conducted, and the average results of each fold are taken as the final results.

### 3.2. Evaluation Criterion

In order to measure and compare effectively and comprehensively the performance of ML-kNN, multiple evaluation criterions are computed, including Precision, Macroaverage F1-Measure, Microaverage F1-Measure, Coverage, Hamming Loss, One Error, and Ranking Loss. Each criterion has its own characteristic which display one aspect of a model's performance. More information about these criterions can be found in [9].

### 3.3. Experimental Results and Discussions

Table 6 summarizes the experimental results on the five single-diagnosis datasets and the one fusional-diagnosis dataset. All the seven evaluation criterions are configured to be the bigger the better, even for negative number (the closer to zero, the better).

From the Table 6, we can find the following.

1. The model built on inspection-diagnosis dataset performs the best in all the evaluation criterions, among the 5 models built on single-diagnosis datasets, which demonstrates that inspection may be the best way to differentiate ZHENG about hypertension.
2. For all evaluation criterions, performance of fusional-diagnosis model is the best, which may prove strongly the TCM theory that “fusion use of the four classical diagnostic methods” is essential and help improve the accuracy of ZHENG differentiation.

## 4. Conclusions 

In this paper, we attempted to use feature-level information fusion technique and ML-kNN algorithm to improve performance of intelligent ZHENG classification, which is a tough but essential task in TCM. Instead of using traditional learning methods, according to the characteristics of TCM clinical cases, a popular multilabel learning method, ML-kNN, is used as the classification model. Information fusion to properly combine information from different diagnostic methods is used to improve classification performance, which confirms the TCM theory of “comprehensive analysis of data gained by four diagnostic methods.”

In future, we will continue this study to solve the imbalance in the data set and try model level information fusion. 

## Figures and Tables

**Table 1:** Information from inspection diagnosis.

Pale whit complexion	Lusterless complexion	Sallow complexion	Reddened complexion	Bleak complexion	Facial hot flashes	Flushed complexion
Hot eyes	Blue lips	Dark purple lips	Lusterless lips	Red ear	Reddish urine	Yellow urine

Clear abundant urine	Lassitude of spirit	No desire to speak	Listlessness	Palpitate with fear	Impatient	Irritability

**Table 2:** Information from tongue diagnosis.

Pale tongue	Red tongue	Dark red tongue	Pale red tongue	Crimson tongue	Teeth-marked tongue	Tender tongue
Tender and red tongue	Bluish purple tongue	Enlarged and pale tongue	Red margins and tip of the tongue	Petechial on tongue	Enlarged tongue	Dark tongue body

Sublingual collateral vessels tongue	Thin fur	Yellow fur	White slimy fur	Few fur	White fur	Thin yellow fur

Yellow slimy fur	No fur	Thin white fur	Slimy fur	Thick slimy fur	White slippery fur	

**Table 3:** Information from inquiry diagnosis.

Headache	Dizzy	Swelling pain of head-eye	Vertigo	Wrapped head	Heavy-headedness	Stretching
Empty pain	Dizzy vision	Visual deterioration	Blurred vision	Dry	Eyes bulge	Deaf

Tinnitus	Chest pain	Distending pain in hypochondrium	Soreness of waist	Weakness of knees	Oppression in chest	Stuffiness in chest

Weakness of limb	Abdominal distention	Numbness	Anorexia	Dry mouth	Insomnia	Dreamy

Bitter taste in mouth	Bland taste in the mouth	Somnolence	Constipation	Short urine	Frequent nocturia	Sloppy stool

Heat in the palms and soles	Torrid	Cold body	Cold limbs	Fear of cold	Exing heat in the chest palms and soles	

**Table 4:** Information from palpation diagnosis.

Fine	Rough	Fine rapid	Slippery wiry	Fine rapid wiry	Slippery	Weak
Fine wiry	Rough wiry	Slippery rapid	Rapid	Intermittent bound	Soggy slippery	Rapid wiry

Wiry	Fine weak	Rough sunken	Fine wiry	Soggy	Fine rough	Fine sunken

**Table 5:** Information from other diagnosis.

Night sweating	Palpitate	Muscular twitching and cramp	Sputum	Facial paralysis	Spermatorrhoea	Palpitation
Nausea vomiting	Dry in the throat	Stiffness of the neck	Forgettery	Short breath	Lusterless of hair	Luxated tooth

Heavy body	Impotence	Shortness of breath	Retch nausea sputum	Fat		

**Table 6:** Experimental results of ML-kNN on Six datasets.

Dataset type	Inspection	Tongue	Inquiry	Palpation	Others	Fusional
Average precision	0.80	0.77	0.79	0.78	0.77	0.81
Coverage	0.42	0.40	0.41	0.42	0.39	0.44
Hamming loss	−0.13	−0.13	−0.13	−0.13	−0.13	−0.14
macroF1 measure	0.01	0.01	0.00	0.00	0.00	0.01
microF1 measure	0.01	0.01	0.00	0.00	0.01	0.01
One error	−0.34	−0.38	−0.35	−0.38	−0.38	−0.32
Ranking loss	−0.28	−0.31	−0.29	−0.29	−0.32	−0.25
